# READY, FIRE, AIM: DOES INTEGRATING ACUTE AND LONG-TERM SERVICES WORK?

**DOI:** 10.1093/geroni/igac059.745

**Published:** 2022-12-20

**Authors:** Robert Applebaum, Richard Browdie

## Abstract

Due to the increasing costs of Medicaid and Medicare and concerns about how these two programs fail to work together to deliver quality care, there has been a growing enthusiasm for integrated care programs. The Financial Alignment Initiatives (FAI), implemented by the Centers for Medicare and Medicaid Services (CMS) in 2011 and tested in 13 states were designed to test the impacts of a program that offers Medicare and Medicaid services under one organization to individuals who are dually eligible for both programs. Previous studies of the expansion of managed long-term services have generated considerable interest over the last two decades however, research results have been mixed. There is also limited information about the implementation of these efforts, as demonstrations have served varying target populations with very different intervention strategies. The lack of conclusive results means that states, now faced with decisions about continued implementation of these initiatives do not have good information to make sound policy decisions. The national evaluation of the FAI states did not include Medicaid costs. Our study is designed to gain a better understanding of Ohio’s FAI MyCare Demonstration. This symposium provides data from a comprehensive impact analysis that examined both Medicaid and Medicare claims data using a difference- in- differences treatment and comparison analysis (n= 390,000) and an in-depth process evaluation (using interviews with 487 participants) to gain an understanding of program effects. After reviewing results the symposium will discuss the future of these and other reform efforts to integrate Medicaid and Medicare services.

